# Data on mopane worm (*Imbrasia belina*) microorganisms from Limpopo Province, South Africa

**DOI:** 10.1016/j.dib.2020.105695

**Published:** 2020-05-13

**Authors:** Martin J. Potgieter, Naledzani Ramalivhana

**Affiliations:** Department of Biodiversity, University of Limpopo, Private Bag X1106, Sovenga, South Africa

**Keywords:** Edible insects, Health and safety, Mopane worms, Microbes

## Abstract

Mopane worm samples, obtained from different districts of the Limpopo Province of South Africa between April and July 2018, were investigated for the incidence of microbial flora. Over a period of ten weeks, samples consisting of market-obtained, field-prepared and sun- dried worms, were evaluated. It was revealed that after cooking at 89- 93°C, the microbial population was reduced to less than 9 000 CFU/g. The most prominent microbial populations cultured after cooking were spore- formers. Field-processed worms had a microbial population of 4 × 104 -1 × 108 CFU/g. Bacteria and fungi isolated included *Aspergillus niger, Enterobacter aglomerans, Escherichia coli, Micrococcus luteus* and *Penicillium* sp. Total microbial population ranged between 4 × 105 and 3 × 105 CFU/g after cooking. Total bacterial count increased in the sun- dried worms from 3 × 105-4 × 105 CFU/g, while it decreased from 2.0 × 105-1.4 × 105 CFU/g after cooking. This data can be used to generate safety guidelines related to the processing of edible insects, such as mopane worms.

Specifications Table

**Table:** 

Subject	Biology
Specific subject area	Biodiversity and Public Health
Type of data	Table, Graph
How data were acquired	Heterotrophic bacteria was cultivated on trypticase-soya-agar, while fungi were cultivated on Sabouraud-glucose-agar, supplemented with chloramphenicol.
Data format	Raw and Analyzed
Parameters for data collection	Material collected from the field was handled with latex gloves to reduce opportunistic contamination resulting from handling by the research team. In the laboratory the material was processed according to standard techniques.
Description of data collection	Identification of acterial strains were done using commercially available assay kits, such as API 20 NE, API 20E, API 50 CHB and API STAPH. Identification of fungal isolates was morphologically assessed using a Leitz Diaplan microscope, which was equipped with differential interference contrast optics.
Data source location	Institution: University of Limpopo Region: Limpopo Province Country: South Africa
Data accessibility	All data associated with this article are hosted within the article and as a supplementary file

## Value of the data

• This data presents information on the microbiological quality of mopane worms from different locations of the Limpopo Province, South Africa that can be used to generate safety guidelines related to the processing of edible insects.
• The dataset will be of value to the provincial Department of Health in educating street vendors, who sell this product to the general public, on hygienic processing applications.

## 1 Data

### 1.1 Overall counts linked to points-of-collection

**Fig. 1:**
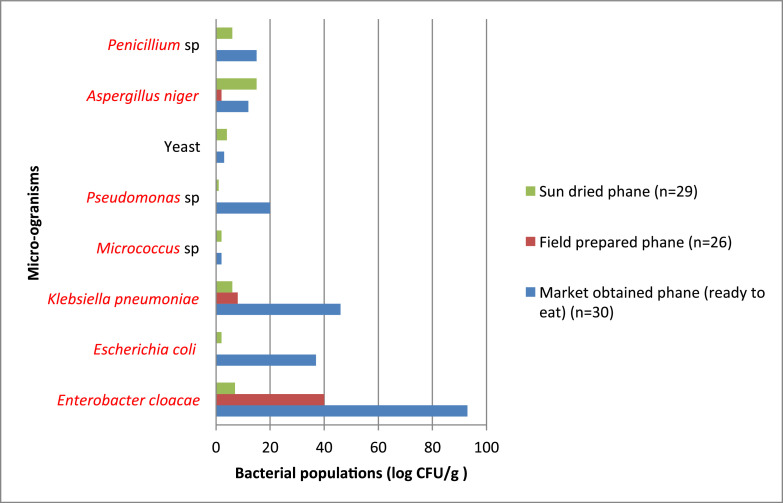
Population of microorganisms isolated from sun-dried, field-prepared and market- obtained worms.

Fig. 1 summarizes the micro-organism count from sun-dried, field-prepared and market obtained mopane worms. Overall, market-obtained samples had the highest associated microbial population. In contrast, field-prepared samples had the lowest microbial population, with the exception of *Enterobacter cloacae* and *Klebsiella pneumonia*.

Data from field-processed worms indicate that the total aerobic mesophilic count of the field- processed mopane worms after cooking ranged from 3 000–9 000 CFU/g (Table 1). The materials left to dry in the field for a day had microbial populations in the range of 50 000–5 × 108 CFU/g (Table 1). This showed an increase in population by a magnitude of 1 to 5 orders. Data from market-obtained worms show that the total aerobic counts (2 × 104–2 × 108 CFU/g) of the worms from the markets were similar to those of the worms from the field (Table 1). The population of the coliform group from the market was much higher than in field processed worms.

**Table 1:** Microbial populations of the field-obtained and market-obtained worms.

Sample	Population of microorganisms in worms (CFU/g)a	
	Field-obtained worms	Market-obtained wormsc
Soon after cooking	3 000–9 000	0
Drying for 1 day	50 000–5 × 108	0
Drying for 2 days	60 000–3 × 108	0
Dried for 3 to 5 days to months	20 000–2 × 108	20–2 × 108
Total coliform countb	0–1 000	0–1 000 000
Yeasts	10–2 000	100–2 000
Molds	Present	Present

aValues represent averages for 6 samples of field processed and 26 samples of market worms.

bTotal coliforms on the worms from the field were estimated after 3—5 days of drying.

cMarket-obtained worms were between 5 days and 2 years old.

### 1.2 Prevalence of coliforms

**Fig. 2:**
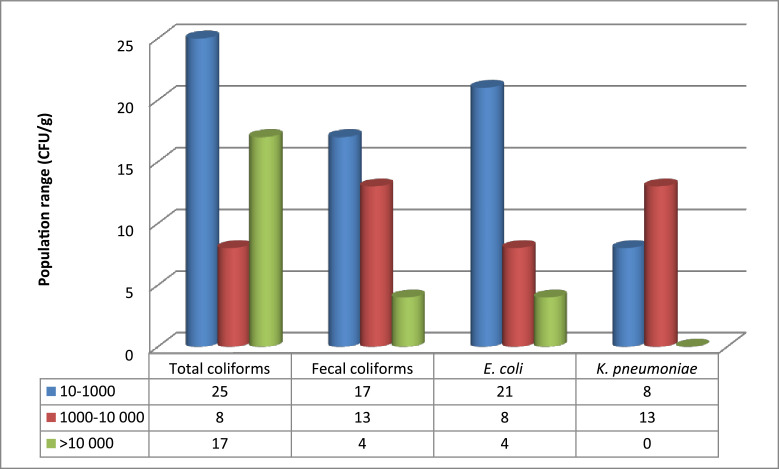
Percentage of samples positive for coliforms in market-obtained worms, by population range.

The population of coliforms in the larvae was high (Fig. 2). However, after cooking field- processed mopane worms were free from coliforms. *Escherichia coli* and *K. pneumoniae* were found in 36% of field-processed and 24% of the market-obtained samples (Fig. 2). In approximately 11% of the samples, *E. coli* was present at levels equal to or exceeding 2 × 104/g. The most abundant of the coliforms were species of *Enterobacter.*

## 2 Experimental Design, Materials, and Methods

Three types of samples of mopane worms were collected between April and July 2018 in the Limpopo Province, South Africa. The samples included 2–3 kg of market-obtained mopane worms (ready-to-eat mopane worms (n=30, worms were randomly selected from this batch), purchased from street vendors; 2–3 kg of field-prepared mopane worms (n=26), which were collected immediately after cooking, and also a 2–3 kg sun-dried mopane worms (n=29). The samples were transferred into a Whirl-Pak bag (Nasco, USA), combined with 180 ml diluent (0.1% Bacteriological Peptone {BioLab, Midrand, South Africa} + 0.85% sodium chloride

{Saarchem-Merck Chemicals, South Africa}). The worms were grounded into powdered form after being washed (distilled water) and air dried in a sterile laminar flow chamber.

### 2.1 Sample processing and enumeration

From each of the samples, 20 g of the powdered worms was transferred into 0.1% Bacteriological Peptone (BioLab, Midrand, South Africa) + 0.85% sodium chloride (Saarchem- Merck Chemicals, South Africa}), and homogenized for 2 min with a Colworth 400 Stomacher. The homogenized samples were serially diluted in diluent, where after they were plated in duplicate, using standard plating procedures.

### 2.2 Isolation of microorganisms

The isolation procedure for all samples was performed in a laminar-flow cabinet and manual operations were conducted using sterile disposable latex gloves. For heterotrophic bacteria, two to three drops from each worm sample was placed on trypticase-soya-agar. For fungi, two to three drops of the worm sample were placed on Sabouraud-glucose-agar that was supplemented with chloramphenicol.

### 2.3 Characterization of bacteria

The bacterial strains, isolated from the prepared samples, were cultured on trypticase-soya- agar. This was done at 30 to 37°C for 48 hours, whilst the replicates were preserved at 4°C using trypticase-soya broth liquid medium that was supplemented with glycerol. Gram staining was done using exponential trypticase-soya-agar cultures. Further investigations included oxidase and catalase tests, acid production from glucose and sucrose using oxidative/fermentative basal medium, their growth on Simmons citrate agar, and the hydrolysis of starch and gelatin.

The ability to form spores was tested in all Gram-positive and certain Gram-negative strains. This was done using the Schaeffer-Fulton specific strain and trypticase-soya-agar and testing at 24 hours, 48 hours, 72 hours and one week intervals [1]. Morphological characters, aimed at viewing high contrast images, were achieved via the combination of phase and differential interference techniques.

Following preliminary morphological and physiological characterization [2], further identification of bacterial strains were done using commercially available assay kits, such as API 20 NE, API 20E, API 50 CHB and API STAPH. In all preparation and inoculation procedures the manufacturer's recommendations (BioMerieux Espana S.A.) were adhered to. Once numeric profiles were obtained they were compared to a bacterial database utilising APILab (BioMerieux, Marcy l'Etoile, France). Simultaneously, additional diagnostic tests were done with some bacterial strains, such as growth on McConkey or mannitol salt agar and coagulase and lysostaphin tests.

### 2.4 Isolation and characterization of fungi

In isolating micromycetes, film samples were incubated on potato-dextrose-agar at 28°C for a period of 1–2 weeks. Replicates were preserved in potato-dextrose broth medium which was supplemented with 10% glycerol and kept at 4°C. For characterisation the following culture media were used:

i Malt extract agar: 20 g malt extract (Difco), 1 g peptone, 20 g glucose, 20 g agar and 1L distilled water;
ii Czapek yeast extract agar: 1 g dipotassium hydrogenphosphate, 10 ml Czapek concentrate, 5 g yeast extract (Difco), Sucrose 30 g, 15 g agar and 1L distilled water;
iii 25% glycerol nitrate agar: 0.75 g di potassium hydrogenphosphate, 7.5 ml Czapek concentrate, 3.7 g yeast extract, glycerol (analytical grade) 250 g, 12 g agar and 1L distilled water;
iv Potato-carrot agar: 20 g shredded potato, 20 g shredded carrot, 20 g agar and 1 L distilled water.

Seven-day cultures on Malt extract agar, Czapek yeast extract agar (with either 3% or 20% sucrose), and 25% glycerol nitrate agar were used for identification of *Aspergillus* and *Penicillium* species [3,4]. The remainder of the strains were cultured on Potato dextrose agar and Potato-carrot agar for a period of 21 days, at 22°C and 80% humidity. Following this, and in an effort to simulate sporulation, the cultures were subjected to cycles of NUV wavelength light exposure-darkness (12 h each) at room temperature for a minimum of 14 days. The isolates were morphologically assessed using a Leitz Diaplan microscope, which was equipped with differential interference contrast optics. The fungal isolates were identified as either *Aspergillus* or *Penicillium*. Yeast identification was done based on metabolic/physiological features using the API 20CAUX kit (BioMerieux Espana, S.A.). Furthermore, size and shape observations were done after simple crystal violet staining.

## 3 The impact of sodium chloride on growth

In the evaluation of salt tolerance, the isolates were plated by the streak plate technique, in triplicate and on four separate occasions, onto trypticase-soya-agar plates. These agar plates were supplemented with varying sodium chloride concentrations (5, 10, 15, 20, 25%) (Saarchem, Merck Chemicals-South Africa). The inoculated plates were incubated at 37°C, and qualitatively inspected every 24 h up to 7 days for signs of bacterial growth.

## 4 The impact of temperature on bacterial growth

Each isolate was inoculated into 20 ml of trypticase-soya-agar broth, as well as plated by the streak plate technique onto trypticase-soya-agar plates. This was done in triplicate and on four separate occasions. These samples were incubated at 4, 25, 30, 37 and 45°C for 7 days. Bacterial growth on the trypticase-soya-agar plates was assessed at 24 hours intervals. In addition, a loopful from each inoculated trypticase-soya-broth was streak plated onto trypticase-soya-agar plates and incubated for 24 hours at the appropriate temperature. These plates were also observed to confirm any bacterial growth.
